# Supplementary material for: Factors associated with prolonged hospitalizations for COVID-19 during the first three waves of the pandemic: Evidence from a Southeastern State of Brazil
Source: PLoS One. 2025 Sep 9;20(9):e0332128. doi: 10.1371/journal.pone.0332128 (PMC12419605; doi:10.1371/journal.pone.0332128)
Supplement: S1 File — (PDF) [file pone.0332128.s001.pdf]

Métodos and Statistical Analysis

<u>Definição operacional dos desfechos:</u>

A internação de curta e longa duração nas três ondas foi definida com o ponto de corte de 7 dias. Sendo as internações de curta duração com o tempo de duração até 7 dias e as internações de longa duração foram aquelas com 8 dias ou mais.

<u>Definição operacional das exposições:</u>

<u>Características sociodemográficas:</u> faixa etária (até 59 anos; 60 ou mais); sexo (masculino; feminino); anos de estudo (menos que 8 anos; 8 a 11 anos e 11 ou mais anos de estudo).

<u>Número de sintomas:</u> foi definido com base na presença de um ou mais dos seguintes sintomas: febre, dificuldade de respirar, batimento de asa de nariz, tiragem intercostal, cianose, saturação de oxigênio <95%, coma, tosse, produção de escarro, congestão nasal ou conjuntival, coriza, dor na garganta, dificuldade para engolir, diarreia, náusea/vômito, cefaleia, irritabilidade/confusão, adinamia (fraqueza), exsudato faríngeo, conjuntivite, convulsão, perda de olfato, perda de paladar.

<u>Número de comorbidades:</u> o escore foi definido com base na presença de uma ou mais das seguintes comorbidades: doença pulmonar crônica, doença cardiovascular crônica, doença renal crônica, doença hepática crônica, diabetes mellitus, infecção por HIV, neoplasias, cirurgia bariátrica, obesidade, tuberculose, doença neurológica crônica ou neuromuscular.

Análises estatísticas

As análises foram feitas usando o programa estatístico Stata versão 15.0. Realizou-se análises descritivas do banco de dados com frequências brutas, relativas e intervalo de confiança. As análises bivariadas foram realizadas usando teste qui-quadrado de heterogeneidade. Posteriormente, foram calculadas a Razão de Odds / *Odds Ratio* (RO/OR), brutas e ajustadas e seus respectivos intervalos de confiança de 95% (IC 95%) pelo modelo de regressão logística. Todas as variáveis com valor-p<0,20 foram incluídas no modelo com o objetivo de controle de confusão, baseando em modelo conceitual prévio, e estes foram os únicos critérios de seleção de possíveis fatores de confusão a serem ajustados. O nível de significância adotado foi de 5%.

Resultados

Onda 1

Figura 1. Histograma dos dias de internação da onda 1 de Covid do estado do Espírito Santo. Espírito Santo, ES, Brasil (N=2864).

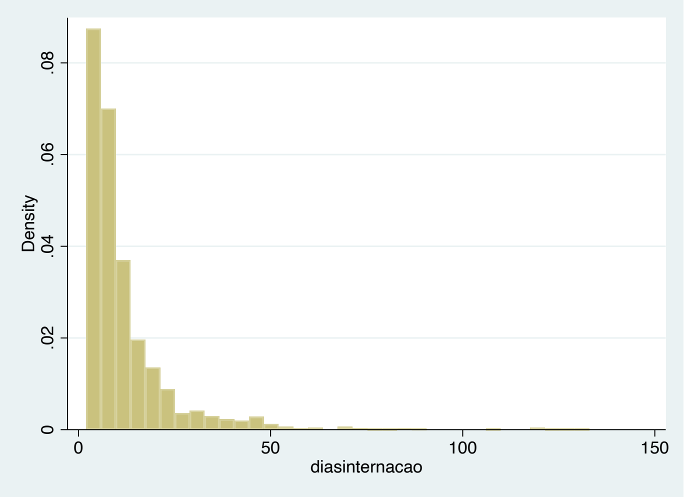

diasinternacao

	Percentiles	Smallest		
1%	2	2		
5%	2	2		
10%	3	2	Obs	2,864
25%	5	2	Sum of Wgt.	2,864
50%	8		Mean	11.5426
		Largest	Std. Dev.	12.66024
75%	14	121		
90%	24	123	Variance	160.2818
95%	35	126	Skewness	3.791081
99%	62	133	Kurtosis	25.14888

Conforme evidencia-se na Figura 1, a distribuição do número de dias de internação é assimétrica à direita (positiva).

Quadro 1. Distribuição do número de dias de internação.

Dias de internação (N=2864)	N (%)	IC95%
Até 7 dias (curta)	1401 (48,9)	47,1-50,7
8 ou mais (longa)	1463 (51,1)	49,3-52,9

N = frequência bruta.
% = frequência relativa.
IC95% = intervalo de confiança de 95%.

Na onda 1 de Covid-19 no estado do Espírito Santo, 51,1% tiveram internações com 8 ou mais dias de duração, considerada longa aqui (Quadro 1).

Características sociodemográficas

Quadro/ Tabela 2. Perfil da amostra, distribuição das características sociodemográficas dos pacientes notificados e hospitalizados por Covid-19 na primeira onda com curta duração e longa duração no Espírito Santo, ES, Brasil. Espírito Santo, ES, Brasil (N = 2864).

Características sociodemográficas	Amostra			Curta duração (Até 7 dias)			Longa duração (8 dias ou mais)				
	N	%	IC95%	N	%	IC95%	N	%	IC95%	x2	Valor-p
**Faixa etária (N=2864)**										46,2	<0,001
Até 59 anos	1372	47,9	46,1-49,7	762	54,4	51,8-57,0	610	41,7	39,2-44,2		
=>60	1492	52,1	50,3-53,9	639	45,6	43,0-48,2	853	58,3	55,8-60,8		
**Sexo (N=2864)**										2,51	0,113
Masculino	1306	45,6	43,8-47,4	660	47,1	44,5-49,7	646	44,2	41,6-46,7		
Feminino	1558	54,4	52,6-56,2	741	52,9	50,3-55,5	817	55,8	53,3-58,4		
**Raça/cor (N=2283)**										0,79	0,373
Brancos	917	40,2	38,2-42,2	427	39,2	36,4-42,1	490	41,0	38,3-43,9		
Não brancos	1366	59,8	57,8-61,8	662	60,8	57,9-63,6	704	59,0	56,1-61,7		

**Anos de estudo completo (N=1625)**										5,73	0,057
Menos que 8	758	46,7	44,2-49,1	344	43,7	40,2-47,1	414	49,5	46,1-52,8		
8 anos até 11	530	32,6	30,4-34,9	268	34,0	30,8-37,4	262	31,3	28,2-34,5		
12 ou mais	337	20,7	18,8-22,8	176	22,3	19,6-25,4	161	19,2	16,7-22,0		

N: frequência bruta.
%: frequência relativa.
IC95%: intervalo de confiança de 95%.
X2: valor do teste qui-quadrado de heterogeneidade.

Na onda 1, a maioria dos pacientes hospitalizados tinham 60 anos ou mais (52,1%), eram do sexo feminino (54,4%), não brancos (60%) e com menos de 8 anos de estudo (Tabela 2). A faixa etária esteve associada a internação de longa duração. Os idosos (60 ou mais anos) apresentaram maior ocorrência de internação de longa duração (Tabela 2).

Regressão Logística

Quadro/Tabela 3. Análise Bruta e Ajustada da Chance de hospitalização por Covid-19 com longa duração e características sociodemográficas. Espírito Santo, ES, Brasil.

Características sociodemográficas	Longa duração					
	Análise bruta			Análise ajustada		
	OR	IC95%	Valor-p	OR	IC95%	Valor-p
**Faixa etária**			<0,001			<0,001a
Até 59 anos	Ref.			Ref.		
=>60	1,67	1,43-1,93		1,67	1,36-2,06	
**Sexo**			0,113			-
Masculino	Ref.			-		
Feminino	1,13	0,97-1,31		-		
**Raça/cor**			0,373			-
Brancos	Ref.			-		
Não brancos	0,93	0,78-1,10		-		

**Anos de estudo completo**			0,057			-
Menos que 8	1,31	1,02-1,70	0,037	-		
8 anos até 11	1,07	0,81-1,40	0,634	-		
12 ou mais	Ref.			-		

OR = Razão de Odds / Odds Ratio.
IC95% = intervalo de confiança de 95%.
Ref. = grupo de referência.
Nota valor-p da análise ajustada: a- condicionado para sexo e anos de estudo.

Na análise ajustada, evidencia-se que a chance de hospitalização de longa duração em pacientes idosos (60 anos ou mais) foi 67% maior quando comparados aos pacientes com até 59 anos, ajustando para sexo e anos de estudo (OR 1,67; IC95% 1,36-2,06, p<0,001) (Tabela 3).

Número de Sintomas

Número de Sintomas (Febre, dificuldade de respirar, batimento de asa de nariz, tiragem intercostal, cianose, saturação de oxigênio <95%, coma, tosse, produção de escarro, congestão nasal ou conjuntival, coriza, dor na garganta, dificuldade para engolir, diarreia, náusea/vômito, cefaleia, irritabilidade/confusão, adinamia (fraqueza), exsudato faríngeo, conjuntivite, convulsão, perda de olfato, perda de paladar

Figura 2. Distribuição / Histograma do número de sintomas da primeira onda de Covid no estado do Espírito Santo. Espírito Santo, ES, Brasil.

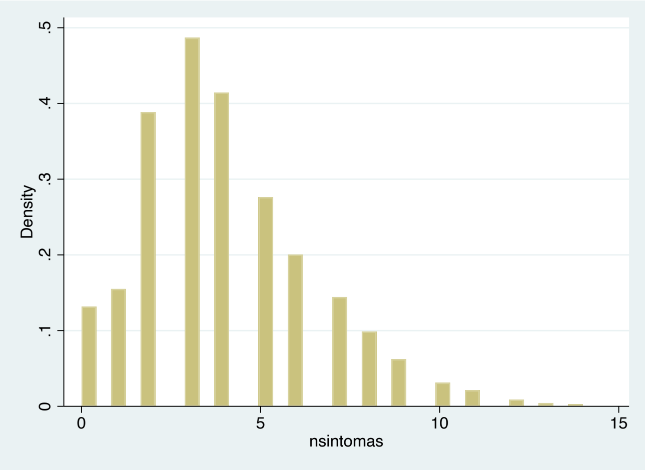

Figura 3. Média, Mediana, Desvio padrão, Skewness e Kurtosis do número de sintomas da primeira onda de Covid no estado do Espírito Santo. Espírito Santo, ES, Brasil.

nsintomas

	Percentiles	Smallest		
1%	0	0		
5%	0	0		
10%	1	0	Obs	2,724
25%	2	0	Sum of Wgt.	2,724
50%	4		Mean	4.015051
		Largest	Std. Dev.	2.439001
75%	5	13		
90%	7	14	Variance	5.948727
95%	9	14	Skewness	.7837741
99%	11	14	Kurtosis	3.670567

A figura 2 e 3 mostram que a distribuição do número é um pouco assimétrica, mas é bem perto da normal, se aproxima muito. Mesmo assim, categorizei/transformei em uma variável categórica ordinal.

Quadro/ Tabela 4. Distribuição dos sintomas da amostra e nos pacientes notificados e hospitalizados por Covid-19 na primeira onda com curta duração e longa duração. Espírito Santo, ES, Brasil.

Sintomas	Amostra			Curta duração			Longa duração				
	N	%	IC95%	N	%	IC95%	N	%	IC95%	x2	Valor-p
**Número de sintomas**										12,11	0,007
0 a 1 sintoma	322	11,8	10,7-13,1	177	13,5	11,2-15,4	145	10,3	8,8-12,0		
2 a 4 sintomas	1447	53,1	51,2-55,0	667	50,8	48,1-53,5	780	55,2	52,6-57,8		
5 a 9 sintomas	878	32,2	30,5-34,0	438	33,4	30,9-36,0	440	31,2	28,8-33,6		
10 ou mais sintomas	77	2,8	2,3-3,5	30	2,3	1,6-3,3	47	3,3	2,5-4,4		

N = frequência bruta.

% = frequência relativa.

IC95% = intervalo de confiança de 95%.

X2 = valor do teste qui-quadrado de heterogeneidade.

Na onda 1 de Covid-19, a maioria dos indivíduos apresentaram de 2 a 4 sintomas (53,1%). Apesar do valor-p dizer que a internação de longa duração esteve associada ao número de sintomas ($p<0,05$), os intervalos de confiança se sobrepõem e não é possível fazer tal afirmação pelos resultados apresentados na tabela 4.

Regressão Logística

Quadro/ Tabela 5. Análise bruta e ajustada da chance de internação de longa duração na primeira onda de Covid-19 no estado do Espírito Santo e número de sintomas. Espírito Santo, ES, Brasil.

Sintomas	Longa duração					
	Análise bruta			Análise ajustada		
	OR	IC95%	Valor-p	OR	IC95%	Valor-p
**Número de sintomas**			0,007			0,056
0 a 1 sintoma	Ref.			Ref.		
2 a 4 sintomas	1,43	1,12-1,82	0,004	1,41	0,95-2,07	0,085
5 a 9 sintomas	1,23	0,95-1,58	0,119	1,15	0,78-1,71	0,483
10 ou mais sintomas	1,91	1,15-3,18	0,012	1,99	1,03-3,83	0,040

OR = Razão de Odds / Odds Ratio.

IC95% = intervalo de confiança de 95%.

Ref. = grupo de referência.
Nota valor-p da análise ajustada: condicionada para faixa etária, sexo, anos de estudo, tabagismo e número de comorbidades.

De acordo com a tabela 5, percebe-se que a chance de internação de longa duração foi 1,99 vezes maior em indivíduos com 10 ou mais sintomas quando comparados aos indivíduos que apresentaram até 1 sintoma, ajustando para faixa etária, sexo, anos de estudo, tabagismo e número de comorbidades (OR 1,99; IC95% 1,03-3,83, p-valor<0,05).

Comorbidades

Número de comorbidades (Doença Pulmonar Crônica, Doença cardiovascular crônica, doença renal crônica, doença hepática crônica, diabetes mellitus, infecção por HIV, neoplasias, cirurgia bariátrica, obesidade, tuberculose, doença neurológica crônica ou neuromuscular).

Quadro/ Tabela 6. Distribuição das comorbidades na amostra e nos pacientes notificados e hospitalizados por Covid-19 na primeira onda com curta duração e longa duração. Espírito Santo, ES, Brasil.

Fatores de risco e Comorbidades	Amostra			Curta duração			Longa duração				
	N	%	IC95%	N	%	IC95%	N	%	IC95%	x2	Valor-p
**Tabagismo (N=2855)**										7,81	0,005
Não	2709	94,9	94,0-95,6	1342	96,1	94,9-97,0	1367	93,8	92,4-94,9		
Sim	146	5,1	4,4-6,0	55	3,9	3,0-5,1	91	6,2	5,1-7,6		
**Obesidade (N=2512)**										34,1	<0,001
Não	2512	88,2	87,0-89,3	1278	91,8	90,2-93,1	1234	84,7	82,8-86,5		
Sim	336	11,8	10,7-13,0	114	8,2	6,9-9,8	222	15,3	13,5-17,2		
**Comorbidades**											
**Número de comorbidades (N=2843)**										85,11	<0,001
Nenhuma	1000	35,2	33,4-36,9	588	42,3	39,7-44,9	412	28,3	26,1-30,7		
1 comorbidade	880	31,0	29,3-32,7	436	31,4	29,0-33,9	444	30,6	28,2-33,0		
2 ou mais comorbidades	963	33,9	32,2-35,6	366	26,3	24,1-28,7	597	41,1	38,6-43,6		

N = frequência bruta.

% = frequência relativa.
IC95% = intervalo de confiança de 95%.
X2 = valor do teste qui-quadrado de heterogeneidade.

Na onda 1 de covid-19 no Espírito Santo, 5,1% dos pacientes internados fumavam, quase 12% eram obesos e cerca de 34% apresentavam 2 ou mais morbidades. O tabagismo, a obesidade e o número de morbidades estiveram associados à internação de longa duração (p<0,05). Os tabagista, obesos e indivíduos com 2 ou mais morbidades apresentaram maior frequência de internação de longa duração (Tabela 6).

Regressão Logística

Quadro/Tabela 7. Análise bruta e ajustada da chance de internação de longa duração de acordo com o tabagismo, obesidade e número de comorbidades na primeira onda de Covid-19 no Espírito Santo, ES, Brasil. Espírito Santo, ES, Brasil.

Fatores de risco e comorbidades	Longa duração					
	Análise bruta			Análise ajustada		
	OR	IC95%	Valor-p	OR	IC95%	Valor-p
**Tabagismo**			0,005			0,068 a
Não	Ref.			Ref.		
Sim	1,62	1,15-2,29		1,51	0,97-2,36	
**Obesidade**			<0,001			<0,001 a
Não	Ref.			Ref.		
Sim	2,02	1,59-2,56		2,23	1,66-2,99	
**Comorbidades**						
**Número de morbidades**			<0,001			<0,001 a
Nenhuma	Ref.			Ref.		
1 morbidade	1,45	1,21-1,74	<0,001	1,34	1,04-1,72	
2 ou mais morbidades (comorbidades)	2,33	1,94-2,79	<0,001	2,23	1,72-2,89	

OR = Razão de Odds/ Odds Ratio.
IC 95% = intervalo de confiança de 95%.
Ref. = grupo referência.

Nota valor-p da análise ajustada: a- ajustado para faixa etária, sexo e anos de estudo.

Conforme visualizado na tabela 7, os indivíduos obesos e com 2 ou mais morbidades apresentaram 2,23 vezes mais chance de internação de longa duração comparados aos indivíduos não obesos e sem morbidades, controlando para possíveis fatores de confusão, essa diferença foi estatisticamente significante ($p<0,05$).

Onda 2

Figura / Histograma 4. Distribuição dos dias de internação da onda 2 de Covid-19 no estado do Espírito Santo. Espírito Santo, ES, Brasil (N=1733).

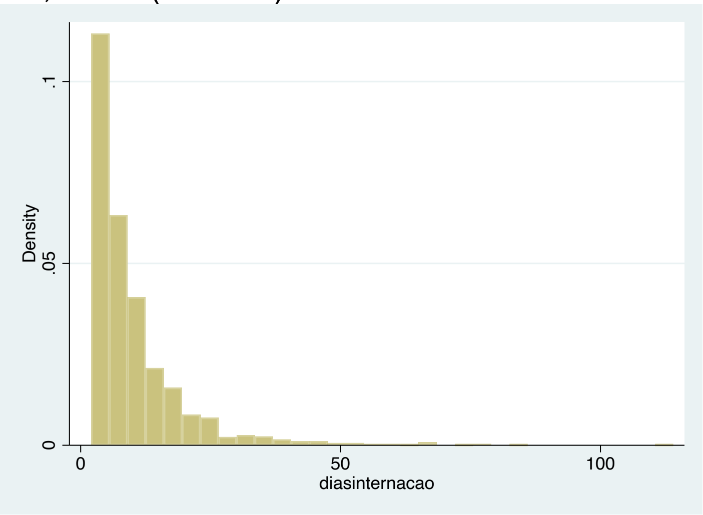

Figura 5. Média, mediana, desvio-padrão do número de dias de internação na segunda onda de Covid-19 no estado do ES.

diasinternacao

	Percentiles	Smallest		
1%	2	2		
5%	2	2		
10%	3	2	Obs	1,733
25%	4	2	Sum of Wgt.	1,733
50%	7		Mean	9.912868
		Largest	Std. Dev.	9.994913
75%	12	76		
90%	21	77	Variance	99.89829
95%	27	85	Skewness	3.317159
99%	52	114	Kurtosis	20.03819

Média 9,9 =~10 dias. Mediana =7 dias. DP = 10. Skewness: 3,3. Kurtosis = 20.

Conforme vemos na figura 4 e 5, a distribuição do número de dias de internação na segunda onda de Covid-19 no estado do ES, é assimétrica à direita.

Quadro 8 . Frequência dos dias de internação da onda 2 de Covid-19 do estado do Espírito Santo. Espírito Santo, ES, Brasil (N=1733).

Dias de internação (N=1733)	N (%)	IC95%
Até 7 dias (curta)	951 (54,9)	52,5-57,2
8 ou mais (longa)	782 (45,1)	42,8-47,5

N = frequência bruta.
% = frequência relativa.
IC95% = intervalo de confiança de 95%.

Conforme relatado anteriormente, optou-se por categorizar todas as ondas com o ponto de corte de 7 dias. No caso da segunda onda, seria a mediana da distribuição do número de dias de internação. Sendo assim, cerca de 45% dos pacientes hospitalizados na segunda onda ficaram internados 8 ou mais dias (longa duração) (Quadro 8).

Características sociodemográficas

Quadro/ Tabela 9. Perfil da amostra, distribuição das características sociodemográficas dos pacientes notificados e hospitalizados por Covid-19 na segunda onda com curta duração e longa duração. Espírito Santo, ES, Brasil.

Características sociodemográficas	Amostra			Curta duração			Longa duração				
	N	%	IC95%	N	%	IC95%	N	%	IC95%	x2	Valor-p
**Faixa etária (N=1733)**										65,80	<0,001
Até 59 anos	761	43,9	41,6-46,3	501	52,7	49,5-55,8	260	33,3	30,0-36,6		
=>60	972	56,1	53,7-58,4	450	47,3	44,2-50,5	522	66,7	63,4-70,0		
**Sexo (N=1733)**										3,66	0,056
Masculino	795	45,9	43,5-48,2	456	47,9	44,8-51,1	339	43,4	39,9-46,9		
Feminino	938	54,1	51,8-56,5	495	52,1	48,9-55,2	443	56,6	53,1-60,1		
**Raça/cor (N=1385)**										0,622	0,430
Brancos	642	46,4	43,7-49,0	345	45,4	41,9-49,0	297	47,5	43,6-51,4		

Não brancos	743	53,6	51,0-56,3	415	54,6	51,0-58,1	328	52,5	48,6-56,4		
**Anos de estudo completo (N=1039)**										24,01	<0,001
Menos que 8	458	44,1	41,1-47,1	205	37,8	33,8-41,9	253	51,0	46,6-55,4		
8 anos até 11	372	35,8	32,9-38,8	230	42,3	38,3-46,6	142	28,6	24,8-32,8		
12 ou mais	209	20,1	17,8-22,7	108	19,9	16,7-24,1	101	20,4	17,0-24,1		

N = frequência bruta.

% = frequência relativa.

IC95% = intervalo de confiança de 95%.

X2 = valor do teste qui-quadrado de heterogeneidade.

Na segunda onda, a maioria dos pacientes tinha 60 anos ou mais, eram do sexo feminino, não brancos e com 8 a 11 anos de estudo. A faixa etária e anos de estudo estiveram associadas a duração da internação por Covid-19. Os indivíduos com 60 anos ou mais e os indivíduos com menor escolaridade tiveram maior ocorrência de longa duração (p<0,001).

Regressão Logística

Quadro/ Tabela 10. Análise Bruta e ajustada . Espírito Santo, ES, Brasil.

**Características sociodemográficas**	**Longa duração**					
	**Análise bruta**			**Análise ajustada**		
	**OR**	**IC95%**	**Valor-p**	**OR**	**IC95%**	**Valor-p**
**Faixa etária (N=1733)**			<0,001			<0,001a
Até 59 anos	Ref.			Ref.		
=>60	2,24	1,84-2,72		2,12	1,63-2,76	
**Sexo (N=1733)**			0,056			-
Masculino	Ref.			-		
Feminino	1,20	1,00-1,46		-		
**Raça/cor (N=1385)**			0,430			-
Brancos	Ref.			-		

Não brancos	0,92	0,74-1,14		-		
**Anos de estudo completo (N=1039)**			<0,001			0,001b
Menos que 8 anos	1,32	0,95-1,83		1,06	0,75-1,49	0,753
8 anos até 11 anos	0,66	0,47-0,93		0,63	0,44-0,89	0,009
12 ou mais	Ref.			Ref.		

OR = Odds Ratio / Razão de Odds.
IC 95% = intervalo de confiança de 95%.
Ref. = grupo de referência.
Nota valor-p da análise ajustada: a- condicionado para sexo e anos de estudo; b – condicionado para faixa etária e anos de estudo.

Os indivíduos com 60 anos ou mais apresentaram 2,12 vezes mais chance de internação de longa duração quando comparados aos indivíduos mais jovens (até 59 anos), controlando para possíveis fatores de confusão (p<0,001). Os indivíduos com 8 a 11 anos de estudo apresentaram 37% menos chance de internação de longa duração do que os indivíduos com 12 ou mais anos de estudo, controlando para possíveis fatores de confusão (p<0,05).

Número de Sintomas

Número de Sintomas (Febre, dificuldade de respirar, batimento de asa de nariz, tiragem intercostal, cianose, saturação de oxigênio <95%, coma, tosse, produção de escarro, congestão nasal ou conjuntival, coriza, dor na garganta, dificuldade para engolir, diarreia, náusea/vômito, cefaleia, irritabilidade/confusão, adinamia (fraqueza), exsudato faríngeo, conjuntivite, convulsão, perda de olfato, perda de paladar.

Figura 6. Distribuição do número de sintomas da segunda onda de Covid-19 do Espírito Santo. Espírito Santo, ES, Brasil.

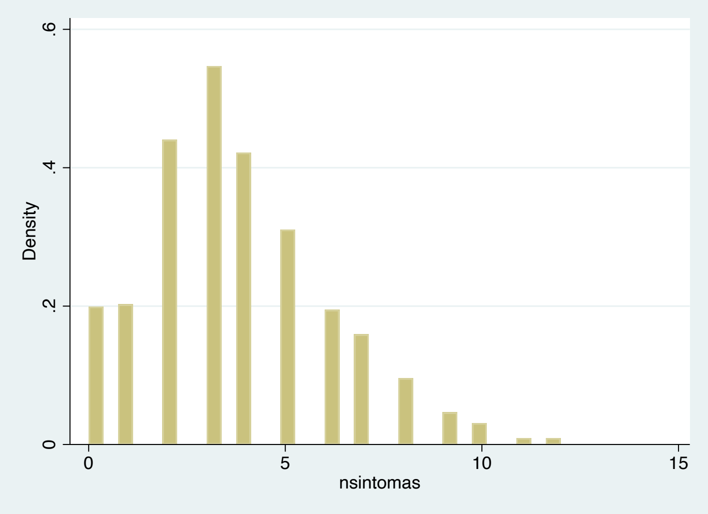

Quadro/ Tabela 11. Distribuição dos sintomas da amostra e nos pacientes notificados e hospitalizados por Covid-19 na segunda onda com curta duração e longa duração. Espírito Santo, ES, Brasil.

Sintomas	Amostra			Curta duração			Longa duração				
	N	%	IC95%	N	%	IC95%	N	%	IC95%	x2	Valor-p
**Número de sintomas (N=1726)**										5,83	0,120
0 a 1 sintoma	260	15,1	13,5-16,8	160	16,9	14,6-19,4	100	12,8	10,7-15,4		
2 a 4 sintomas	912	52,8	50,5-55,2	495	52,2	49,0-55,4	417	53,6	50,0-57,1		
5 a 9 sintomas	522	30,2	28,1-32,5	275	29,0	26,2-32,0	247	31,8	28,6-35,1		
10 ou mais sintomas	32	1,9	1,3-2,6	18	1,9	1,2-3,0	14	1,8	1,1-3,0		

N = frequência bruta.

% = frequência relativa.

IC95% = intervalo de confiança de 95%.

X2 = valor do teste qui-quadrado de heterogeneidade.

<u>Na segunda onda, o número de sintomas não esteve associado à internação de longa duração (p>0,05).</u>

Regressão Logística

Quadro/ Tabela 12. Análise bruta. Espírito Santo, ES, Brasil.

**Sintomas**	**Longa duração**					
	**Análise bruta**			**Análise ajustada**		
	**OR**	**IC95%**	**Valor-p**	**OR**	**IC95%**	**Valor-p**
**Número de sintomas (N=1726)**			0,117			-
0 a 1 sintoma	Ref.					
2 a 4 sintomas	1,35	1,02-1,79	0,038	-	-	
5 a 9 sintomas	1,44	1,06-1,95	0,019	-	-	
10 ou mais sintomas	1,24	0,59-2,61	0,563	-	-	

OR = Odds Ratio / Razão de Odds.
IC 95% = intervalo de confiança de 95%.
Ref. = grupo de referência.

Na análise bruta, indivíduos com 5 a 9 sintomas apresentaram 44% (OR 1,44) mais chance de ter internação de longa duração quando comparados aos indivíduos com até 1 sintoma (p<0,05; IC95% 1,06-1,95).

Comorbidades

Quadro/ Tabela 13. Distribuição das comorbidades da amostra e nos pacientes notificados e hospitalizados por Covid-19 na segunda onda com curta duração e longa duração. Espírito Santo, ES, Brasil.

**Comorbidades**	**Amostra**			**Curta duração**			**Longa duração**				
	**N**	**%**	**IC95%**	**N**	**%**	**IC95%**	**N**	**%**	**IC95%**	**x2**	**Valor-p**
**Tabagismo (N=1732)**										12,65	<0,001
Não	1673	96,6	95,6-97,4	931	98,0	96,9-98,7	742	94,9	93,1-96,2		
Sim	59	3,4	2,6-4,4	19	2,0	1,3-3,1	40	5,1	3,8-6,9		
**Obesidade (N=1730)**										5,10	0,024
Não	1586	91,7	90,3-92,9	882	93,0	91,2-94,5	704	90,0	87,7-91,9		

Sim	144	8,3	7,1-9,7	66	7,0	5,5-8,8	78	10,0	8,1-12,3		
**Comorbidades**											
**Número de morbidades**										53,6	<0,001
Nenhuma	796	46,2	43,9-48,6	507	53,5	50,3-56,7	289	37,3	33,9-40,7		
1 morbidade	452	26,2	24,2-28,4	236	24,9	22,3-27,8	216	27,8	24,8-31,1		
2 ou mais morbidades (comorbidades)	475	27,6	25,5-29,7	204	21,6	19,0-24,3	271	34,9	31,6-38,3		

N = frequência bruta.

% = frequência relativa.

IC95% = intervalo de confiança de 95%.

Na 2ª onda, cerca de 3% eram tabagistas, 8% obesos e 27,6 tinham 2 ou mais morbidades. O tabagismo, obesidade e número de morbidades estiveram associados à duração da internação (p<0,05).

Regressão Logística

Quadro/ Tabela 14. Análise bruta e ajustada. Espírito Santo, ES, Brasil.

**Comorbidades**	**Longa duração**					
	**Análise bruta**			**Análise ajustada**		
	**OR**	**IC95%**	**Valor-p**	**OR**	**IC95%**	**Valor-p**
**Tabagismo**			<0,001			0,061
Não	Ref.			Ref.		
Sim	2,64	1,52-4,60		1,89	0,97-3,97	
**Obesidade**			0,024			0,160a
Não	Ref.			Ref.		
Sim	1,48	1,05-2,09		1,32	0,90-1,96	
**Comorbidades**						
**Número de morbidades**			<0,001			0,001b
Nenhuma	Ref.			Ref.		

1 morbidade	1,61	1,27-2,03	<0,001	1,23	0,90-1,69	0,201
2 ou mais morbidades (comorbidades)	2,33	1,85-2,94	<0,001	1,85	1,34-2,55	<0,001

OR = Odds Ratio / Razão de Odds.
IC 95% = intervalo de confiança de 95%.
Ref. = grupo de referência.
Nota valor-p da análise ajustada: a - condicionada para faixa etária, sexo e anos de estudo; b – condicionada para faixa etária, sexo e anos de estudo e tabagismo.

Após ajustes, os indivíduos com 2 ou mais morbidades apresentaram 85% mais chance de internação de longa duração quando comparados aos sem nenhuma morbidade (p<0,001).

Onda 3

Figura / Histograma 7. Distribuição dos dias de internação por Covid-19 da onda 3 no estado do Espírito Santo. Espírito Santo, ES, Brasil (N=4415).

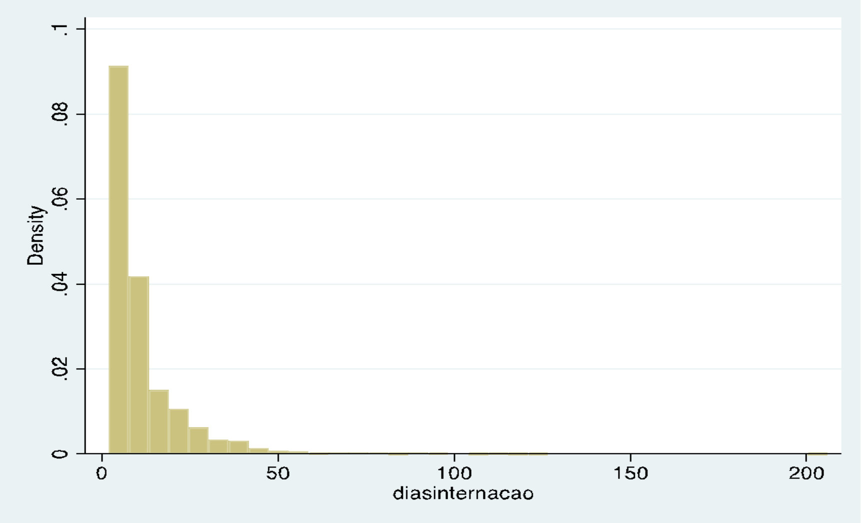

Média = 11,2 =~11 dias. Mediana = 7 dias. DP = 12,2. Skewness = 3,9. Kurtosis = 31,6.

Quadro/Tabela 15. Distribuição dos dias de internação na terceira onda de Covid-19 usando o ponto de corte de 12 dias. Espírito Santo, ES, Brasil.

**Dias de internação (N=4415)**	**N (%)**	**IC95%**
Até 7 dias (curta)	2285 (51,8)	50,3-53,2
8 ou mais (longa)	2130 (48,2)	46,8-49,7

N = frequência bruta.

% = frequência relativa.

IC95% = intervalo de confiança de 95%.

Na 3ª onda, cerca de 48% dos indivíduos tiveram internação de longa duração.

Características sociodemográficas

Quadro/ Tabela 16. Distribuição das características sociodemográficas da amostra e nos pacientes notificados e hospitalizados por Covid-19 na terceira onda com curta duração e longa duração. Espírito Santo, ES, Brasil.

	Amostra			Curta duração			Longa duração				
	N	%	IC95%	N	%	IC95%	N	%	IC95%	x2	Valor-p
**Faixa etária (N=4415)**										94,61	<0,001
Até 59 anos	2591	58,7	57,2-60,1	1500	65,6	63,7-67,6	1091	51,2	49,1-53,3		
=>60	1824	41,3	39,9-42,8	785	34,4	32,4-36,3	1039	48,8	46,7-50,9		
**Sexo (N=4415)**										2,08	0,150
Masculino	2004	45,4	43,9-46,9	1061	46,4	44,4-48,5	943	44,3	42,2-46,4		
Feminino	2411	54,6	53,1-56,1	1224	53,6	51,5-55,6	1187	55,7	53,6-57,8		
**Raça/cor (N=3485)**										2,83	0,092
Brancos	1476	42,4	40,7-44,0	789	43,7	41,4-46,0	687	40,9	38,6-43,3		
Não brancos	2009	57,6	56,0-59,3	1016	56,3	54,0-58,6	993	59,1	56,7-61,4		
**Anos de estudo completo (N=2273)**										2,89	0,236
Menos que 8	1076	47,3	45,3-49,4	522	47,2	44,2-50,1	554	47,5	44,7-50,4		
8 anos até 11	836	36,8	34,8-38,8	422	38,1	35,3-41,0	414	35,5	32,8-38,3		
12 ou mais	361	15,9	14,4-17,4	163	14,7	12,8-16,9	198	17,0	14,9-19,2		

N = frequência bruta.

% = frequência relativa.

IC95% = intervalo de confiança de 95%.

X2 = valor do teste qui-quadrado de heterogeneidade.

Na 3ª onda, a maioria dos indivíduos tinha até 59 anos, eram do sexo feminino, não brancos e com 8 a 11 anos de estudo. A faixa etária esteve associada à duração da internação (p<0,001). Os indivíduos com 60 anos ou mais apresentaram maior frequência de internação (Tabela 16).

Regressão Logística

Quadro/ Tabela 18. Análise Bruta e ajustada. Espírito Santo, ES, Brasil.

Características sociodemográficas	Longa duração					
	Análise bruta			Análise ajustada		
	OR	IC95%	Valor-p	OR	IC95%	Valor-p
**Faixa etária (N=4415)**			<0,001			<0,001a
Até 59 anos	Ref.			Ref.		
=>60	1,82	1,61-2,05		1,89	1,64-2,17	
**Sexo (N=4415)**			0,150			-
Masculino	Ref.			-	-	
Feminino	1,09	0,97-1,23		-	-	
**Raça/cor (N=3485)**			0,092			-
Brancos	Ref.			-	-	
Não brancos	1,12	0,98-1,28		-	-	
**Anos de estudo completo (N=1039)**			0,236			-
Menos que 8	0,87	0,69-1,11		-	-	
8 anos até 11	0,81	0,63-1,03		-	-	
12 ou mais	Ref.			-	-	

OR = Odds Ratio / Razão de Chances.
IC95% = intervalo de confiança de 95%.
Ref. = grupo de referência.
Nota valor-p da análise ajustada: a- condicionado para sexo e raça/cor.

Os indivíduos com 60 anos ou mais apresentaram 89% mais chance de internação de longa duração quando comparados aos indivíduos com idade até 59 anos, ajustando para possíveis fatores de confusão (p<0,001).

Sintomas

Número de Sintomas (Febre, dificuldade de respirar, batimento de asa de nariz, tiragem intercostal, cianose, saturação de oxigênio <95%, coma, tosse, produção de escarro, congestão nasal ou conjuntival, coriza, dor na garganta, dificuldade para engolir, diarreia, náusea/vômito, cefaleia, irritabilidade/confusão, adinamia (fraqueza), exsudato faríngeo, conjuntivite, convulsão, perda de olfato, perda de paladar.

Figura 8. Distribuição do número de sintomas da onda 3 de Covid-19 no estado do Espírito Santo. Espírito Santo, ES, Brasil (N=4415).

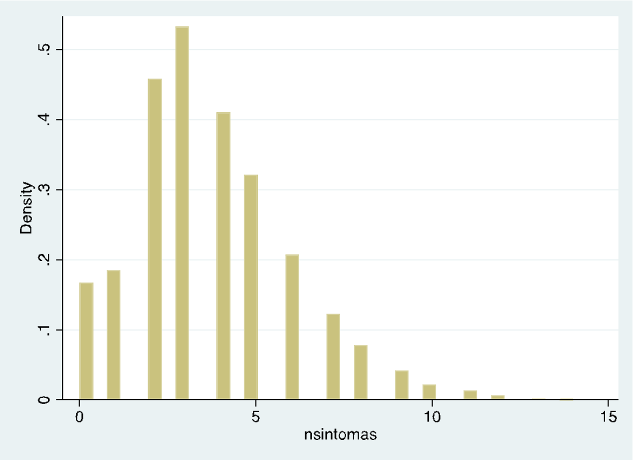

Quadro/ Tabela 19. Distribuição dos sintomas da amostra e nos pacientes notificados e hospitalizados por Covid-19 na terceira onda com curta duração e longa duração. Espírito Santo, ES, Brasil.

Sintomas	Amostra			Curta duração			Longa duração				
	N	%	IC95%	N	%	IC95%	N	%	IC95%	x2	Valor-p
**Número de sintomas (N=4407)**										25,10	<0,001
0 a 1 sintoma	605	13,7	12,7-14,8	344	15,1	13,7-16,6	261	12,3	10,9-13,7		
2 a 4 sintomas	2402	54,5	53,0-56,0	1280	56,2	54,1-58,2	1122	52,7	50,6-54,8		
5 a 9 sintomas	1323	30,0	28,7-31,4	612	26,8	25,1-28,7	711	33,4	31,4-35,4		

10 ou mais sintomas	77	1,8	1,4-2,2	43	1,9	1,4-2,5	34	1,6	1,1-2,2		

N = frequência bruta.
% = frequência relativa.
IC95% = intervalo de confiança de 95%.

Na 3ª onda, a maioria da amostra apresentou 2 a 4 sintomas (54,5%). O número de sintoma esteve associado à internação de longa duração (p<0,05).

Regressão Logística

Quadro/ Tabela 20. Análise bruta e ajustada. Espírito Santo, ES, Brasil.

**Sintomas**	**Longa duração**					
	**Análise bruta**			**Análise ajustada**		
	**OR**	**IC95%**	**Valor-p**	**OR**	**IC95%**	**Valor-p**
**Número de sintomas (N=4407)**			<0,001			0,001
0 a 1 sintoma	Ref.			Ref.		
2 a 4 sintomas	1,16	0,97-1,38	0,115	1,18	0,95-1,46	0,143
5 a 9 sintomas	1,53	1,26-1,86	<0,001	1,50	1,19-1,89	0,001
10 ou mais sintomas	1,04	0,65-1,68	0,866	0,90	0,53-1,51	0,678

OR = Razão de Odds / Odds Ratio.
IC 95% = intervalo de confiança de 95%.
Ref. = grupo de referência.
Nota valor-p da análise ajustada: condicionada para faixa etária, sexo, raça/cor, tabagismo e número de comorbidades.

O número de sintoma esteve associado à internação de longa duração (p<0,05). Após ajustes, os indivíduos com 5 a 9 sintomas apresentaram 50% mais chance de internação de longa duração quando comparados aos indivíduos com até 1 sintoma (p<0,05).

Comorbidades

Número de comorbidades (Doença Pulmonar Crônica, Doença cardiovascular crônica, doença renal crônica, doença hepática crônica, diabetes mellitus, infecção por HIV, neoplasias, cirurgia bariátrica, obesidade, tuberculose, doença neurológica crônica ou neuromuscular).

Quadro/ Tabela 21. Distribuição dos comorbidades na amostra e nos pacientes notificados e hospitalizados por Covid-19 na terceira onda com curta duração e longa duração. Espírito Santo, ES, Brasil.

Comorbidades	Amostra			Curta duração			Longa duração				
	N	%	IC95%	N	%	IC95%	N	%	IC95%	x2	Valor-p
**Tabagismo (N=4409)**										5,87	0,015
Não	4313	97,8	97,3-98,2	2246	98,3	97,7-98,8	2067	97,3	96,5-97,9		
Sim	96	2,2	1,8-2,7	38	1,7	1,2-2,3	58	2,7	2,1-3,5		
**Obesidade (N=4409)**										54.53	<0,001
Não	3995	90,6	89,7-91,4	2141	93,7	92,7-94,7	1854	87,2	85,8-88,6		
Sim	414	9,4	8,6-10,3	143	6,3	5,3-7,3	271	12,8	11,4-14,2		
**Comorbidades**											
**Número de morbidades**										116,1	<0,001
Nenhuma	2447	55,5	54,1-57,0	1434	62,9	60,9-64,8	1013	47,7	45,6-49,8		
1 morbidade	1029	23,4	22,1-24,6	485	21,2	19,6-23,0	544	25,6	23,8-27,5		
2 ou mais morbidades (comorbidades)	930	21,1	19,9-22,3	362	15,9	14,4-17,4	568	26,7	24,9-28,7		

N = frequência bruta.
% = frequência relativa.
IC95% = intervalo de confiança de 95%.

Na 3ª onda, cerca de 2% era tabagista, 9,4% era obeso e 55,5% não tinha nenhuma morbidade. O tabagismo, a obesidade e número de morbidades estiveram associados a duração da internação (p<0,05).

Regressão Logística

Quadro/ Tabela 22. Análise bruta e ajustada. Espírito Santo, ES, Brasil.

	Longa duração					
**Comorbidades**	**Análise bruta**			**Análise ajustada**		
	**OR**	**IC95%**	**Valor-p**	**OR**	**IC95%**	**Valor-p**
**Tabagismo (N=4409)**			0,015			0,200a
Não	Ref.			Ref.		
Sim	1,66	1,10-2,51		1.33	0,86-2,06	
**Obesidade (N=4409)**			<0,001			<0,001b
Não	Ref.			Ref.		
Sim	2,19	1,77-2,71		2,24	1,78-2,82	
**Comorbidades**						
**Número de morbidades**			<0,001			<0,001c
Nenhuma	Ref.			Ref.		
1 morbidade	1,59	1,37-1,84	<0,001	1,50	1,27-1,79	<0,001
2 ou mais morbidades (comorbidades)	2,22	1,90-2,59	<0,001	2,03	1,69-2,43	<0,001

OR = Razão de Odds / Odds Ratio.
IC 95% = intervalo de confiança de 95%.
Ref. = grupo de referência.
Nota valor-p da análise ajustada: a - condicionada para faixa etária, sexo e raça/cor; b – condicionada para faixa etária, sexo e raça/cor e tabagismo; c- condicionada para faixa etária, sexo e raça/cor e tabagismo.

Conforme mostra a tabela 22, após ajustes para fatores de confusão, os indivíduos obesos apresentaram 2,24 vezes mais chance de internação de longa duração quando comparados aos não obesos (OR 2,24; IC95% 1,78-2,82, p<0,001). Condicionando para possíveis fatores de confusão, indivíduos com uma morbidade e com 2 ou mais morbidades apresentaram, 1,5 e 2,0, respectivamente, maior chance de internação de longa duração quando comparados aos indivíduos sem nenhuma morbidade (p<0,001).
