# Supplementary material for: Factors associated with prolonged hospitalizations for COVID-19 during the first three waves of the pandemic: Evidence from a Southeastern State of Brazil
Source: PLoS One. 2025 Sep 9;20(9):e0332128. doi: 10.1371/journal.pone.0332128 (PMC12419605; doi:10.1371/journal.pone.0332128)
Supplement: Attachment — Submitted filename: Response to Reviewers.pdf [file pone.0332128.s003.pdf]

Vitória, February 26th 2025

Response Letter to the Reviewers

Dear Editors and Reviewers,

We sincerely appreciate the opportunity to revise and improve our manuscript based on the insightful comments and constructive suggestions provided by the reviewers. We believe that these recommendations have significantly enhanced the quality and clarity of our study. Below, we provide a detailed point-by-point response to each comment, indicating the revisions made in the manuscript.

Decision: Minor Revision

Reviewer 1:

a) Introduction: The authors of the current manuscript could briefly describe complications of prolonged hospitalization due to COVID-19, such as long-term COVID-19.
Response: Ok. Done! The following sentence was added: *"It is important to highlight that patients with severe manifestations, such as acute respiratory distress syndrome (ARDS) and those requiring intensive care unit (ICU) admission, are more likely to develop long-term sequelae due to the persistence of symptoms, commonly referred to as long COVID-19."*

b) Materials and Methods: In the second paragraph, what do the authors mean about 19?
Response: OK. This was a typographical error. The number was removed.

c) Measures: The authors must report the types and places of hospitalized patients with COVID-19, such as wards or intensive care units.
Response: Both general ward and intensive care unit (ICU) hospitalizations were considered. The following sentence was added: *"Hospitalizations in both general wards and intensive care units (ICUs) were taken into account."*

d) Could the authors report outcomes, such as the need for mechanical ventilation, dialysis, or even mortality, to compare the different waves of the COVID-19 pandemic?
Response: This study relied on secondary data from state notification records. Unfortunately, data on mechanical ventilation and dialysis were not included in these records. Since our objective was to analyze risk factors associated with hospitalization, mortality was not analyzed. However, we have previously published a study on survival analysis (Garbin, J.R.T. et al., *Int. J. Environ. Res. Public Health* 2022, *19*, 8709. https://doi.org/10.3390/ijerph19148709). Future studies may explore this further.

e) Results: Was there any statistical difference in the distribution of hospitalizations between the three waves?
Response: Yes. Table 1 indicates a statistically significant difference in hospitalization

distribution, as shown by the 95% confidence interval, particularly between the first and second waves and the first and third waves. Additional information, including the p-value, has been included in line 149. We also published similar results in another study (Garbin, J.R.T. et al., *Sci Rep* 2024, *14*, 5777. https://doi.org/10.1038/s41598-024-56289-7).

f) Authors need to write better about significant comorbidities, comparing each wave to help understand any changes in comorbidities across these waves.
Response: Thank you for this relevant comment. The text was adjusted for improved clarity.

g) Again, were the authors able to determine outcomes related to the length of hospital stay?
Response: Yes, we observed that prolonged hospitalizations were more frequent during the first wave. The most important risk factors consistently associated with long-term hospitalizations were age (with patients aged 60 years or older more likely to experience prolonged stays), smoking, obesity, and comorbidities. Specifically, in the first wave, prolonged hospitalizations were associated with the number of symptoms; in the second wave, lower educational attainment was associated with longer stays; and in the third wave, symptom burden was again a key factor.

h) Was there a longer length of hospital stay in intensive care units in which wave of the COVID-19 pandemic?
Response: Our study did not separately analyze ICU hospitalizations by wave. Both general ward and ICU hospitalizations were included in our analysis, as our objective was to examine factors associated with short-term and prolonged hospitalization for COVID-19 across the three pandemic waves, regardless of hospitalization type.

i) The authors could describe molecular and pathophysiological mechanisms evident in the literature and explain why some comorbidities are related to a higher risk of prolonged hospitalization due to COVID-19. For example, in the elderly, is reduced innate immunity predisposed to more severe SARS-CoV-2 infection?
Response: Yes, we have added literature describing molecular mechanisms in the discussion, as suggested. Thank you!

Reviewer 2:

1. Define the level of support the state had offered in the acknowledgment section.
Response: The government of Espírito Santo, through the State Health Department, authorized and provided secondary data for this study. This information has been added to the acknowledgment section.

2. Revise English.
Response: The entire manuscript has been revised for English language improvement, as recommended.

3. Improve the introduction section; shed light on the Brazilian COVID-19 experience and Brazilian pandemic mitigation strategies.
Response: We have added a paragraph addressing this aspect.

4. Reflect on the rationale of the study.
Response: We have rewritten the justification to enhance clarity and better reflect the manuscript's content.

5. Revise the discussion section. Link the results to the pandemic management protocol.
Response: The discussion section has been revised, and the results have been linked to Brazil's pandemic management protocols. A paragraph was added at the end of the discussion.

We sincerely appreciate all the comments and suggestions, which have substantially improved our manuscript. Thank you for your time and consideration.

Best regards,
The Authors
