# Supplementary material for: Factors associated with prolonged hospitalizations for COVID-19 during the first three waves of the pandemic: Evidence from a Southeastern State of Brazil
Source: PLoS One. 2025 Sep 9;20(9):e0332128. doi: 10.1371/journal.pone.0332128 (PMC12419605; doi:10.1371/journal.pone.0332128)
Supplement: Attachment — Submitted filename: Response to Reviewers2.pdf [file pone.0332128.s004.pdf]

Vitória, ES, on August 12, 2025

Manuscript title: Factors associated with prolonged hospitalizations for COVID-19 during the first three waves of the pandemic: evidence from a Southeastern state of Brazil

Response Letter to the Associate Editor and Editor-in-Chief of PLOS One,

Editor Comments:

The manuscript "Factors associated with prolonged hospitalizations for COVID-19 during the first three waves of the pandemic: evidence from a Southeastern state of Brazil" presents a retrospective observational study analyzing factors associated with short and prolonged hospitalizations due to COVID-19. Using data from Espírito Santo state in Brazil, the manuscript classified hospitalizations as short or prolonged based on a 7-day threshold. They conducted bivariate analyses using chi-square tests and employed logistic regression models to explore associations between clinical and sociodemographic factors and hospitalization duration across the first three pandemic waves. The study's main finding is that prolonged hospitalizations were more frequent in the first wave. In contrast, shorter stays predominated in the second and third waves, with older age, obesity, symptom burden, and comorbidities associated with prolonged hospitalization.

Given that I have assumed editorial responsibility after prior rounds of peer review, and considering the manuscript has already addressed comments from previous reviewers, I will request only a limited set of fundamental revisions before the manuscript can be considered for acceptance. If these changes are fully addressed, the manuscript will be approved for publication.

Response: We sincerely appreciate the opportunity to revise and improve our manuscript in response to the insightful comments and constructive suggestions provided. We fully agree with the points raised, and we have incorporated all the recommended changes into the manuscript as advised. We are confident that these revisions have enhanced the clarity, robustness, and overall quality of our study.

Below, we provide a detailed point-by-point response to each editorial comment, indicating the specific modifications made in the revised version.

Decision: Minor Revision

1.Empirical Testing of the Main Hypothesis: the manuscript's argument regarding differences in the proportion of prolonged hospitalizations across the three waves is presented emphatically. Still, it lacks formal statistical testing to support the conclusion. I request the authors to perform two additional analyses:

First, a logistic regression model using hospitalization duration (short vs. long) as the

dependent variable and the pandemic wave as the key independent variable for the full dataset, composed of the three waves. A second model using hospitalization duration in days as a continuous dependent variable, preferably applying an appropriate regression model to compare the mean duration across waves. These analyses are essential to substantiate the claim that the profile of hospitalization duration significantly changed across waves. The conclusions about differences between waves should only be presented after conducting and reporting these tests.

Resposta: As recommended, we performed the additional statistical analyses to substantiate our conclusions regarding differences in hospitalization duration across the three pandemic waves. Specifically, we applied a simple robust regression model with robust standard errors to account for potential violations of the homoscedasticity assumption. This approach provides estimates that are more resilient to the presence of outliers and does not require the residuals to follow a normal distribution, which is an important premise in this context.

The assumption of serial autocorrelation was evaluated using the Durbin–Watson test. Multicollinearity was not assessed, as only one independent variable was included in the model. The regression evaluated the association between COVID-19 hospitalization duration and the pandemic wave of occurrence, with the alpha level for statistical significance set at 5%.

For the logistic regression model, the analysis was statistically significant ($p < 0.001$), allowing for valid inferences. The model's explanatory power (pseudo R^2) was 2.0%, indicating that it explained 2.0% of the variation in the dependent variable. The Omnibus test was also significant ($p = 0.001$), confirming that the independent variable influenced the outcome. Furthermore, the Hosmer–Lemeshow test indicated no rejection of the null hypothesis that the model fit the data well ($p = 0.999$). Compared to the first wave, the second wave was associated with a 21.0% lower likelihood of prolonged hospitalization (≥8 days) relative to short hospitalization (≤7 days), while the third wave showed an 11.0% reduction (Table 6).

For the second model, which used hospitalization duration in days as a continuous dependent variable, the regression was also statistically significant ($p = 0.004$), enabling valid inferences. The explanatory power (pseudo R^2) was 2.4%, meaning the model explained 2.4% of the variation in the dependent variable. The Durbin–Watson statistic was close to 2, indicating no evidence of serial autocorrelation. In this model, the second wave of COVID-19 showed a significant trend toward a reduced mean length of stay compared with the first wave (−0.57 days, $p = 0.001$), whereas the third wave was not statistically significant, indicating that the mean hospitalization duration was not influenced by this wave (Table 7).

2) Throughout the manuscript, the authors redundantly report the confidence level (95% CI) and the alpha level (5%) when describing statistical results. It is unnecessary to state both, as a 95% confidence interval inherently implies an alpha of 5%. Please standardize the reporting and remove redundant mentions of the alpha level where confidence intervals are already presented.

Resposta: All redundant mentions of the alpha level were removed in sentences where the 95% confidence interval was already reported. The statistical reporting has been standardized throughout the manuscript to ensure clarity and avoid unnecessary repetition.

3) Definition of Long vs. Short Hospitalizations: the use of 7 days as the threshold to define short and prolonged hospitalizations needs further justification. The current explanation that it corresponds to the median of the second wave is insufficient. Please elaborate on why the second wave median was chosen as the cutoff and discuss its clinical relevance. Additionally, as a robustness check, I recommend testing alternative thresholds (e.g., 6 days or 10 days) and reporting whether the main associations remain consistent.

Resposta: We appreciate this observation. In this study, the classification of short-term and prolonged hospitalizations across the three pandemic waves was based on a 7-day cutoff, corresponding to the median hospital stay observed during the second wave. This parameter was similar to the study by Wu et al. (2020), which also conducted its analyses according to the median hospitalization time, using univariable and multivariable logistic regression methods to identify risk factors associated with long-term hospitalization in patients with COVID-19 [16].

Wu Y, Hou B, Liu J, Chen Y, Zhong P. Risk factors associated with long-term hospitalization in patients with COVID-19: A single-centered, retrospective study. Front Med (Lausanne). 2020;7:315. doi:10.3389/fmed.2020.00315.

In addition, a Brazilian study published in PLOS ONE (de Andrade et al., 2020) reported that the length of hospital stay for COVID-19 patients admitted to the Brazilian Unified Health System (SUS) ranged from less than 24 hours to 114 days, with an average of 6.9 days, which corroborates our findings [17].

de Andrade CLT, Pereira CCdA, Martins M, Lima SML, Portela MC. COVID-19 hospitalizations in Brazil's Unified Health System (SUS). PLoS One. 2020;15(12):e0243126. doi:10.1371/journal.pone.0243126.

Accordingly, the Methods section was updated to read: "Using the median length of stay as a threshold has been reported in the literature as a method to assess hospital course in homogeneous groups with distinct clinical outcomes [16]. Furthermore, the choice of a 7-day cutoff is consistent with national data

indicating an average hospital stay of 6.9 days among patients admitted to public hospitals within the Brazilian Unified Health System (SUS), reinforcing the clinical representativeness of this threshold for our study population [17]."

4) Standardization of Regression Tables: The presentation of regression results should follow a more conventional format. Specifically, remove the reference category rows ("Ref.") as a separate line in the table. Present only the Odds Ratios (OR) and their standard errors. Confidence intervals and p-values should be omitted from the table body, and p-values should be indicated using conventional asterisks (*p<0.05; **p<0.01; ***p<0.001). Consider eliminating the crude (unadjusted) models from the main regression tables and moving them to a statistical appendix if necessary. In the main text, I recommend presenting only the adjusted models, using a clearer layout to improve readability.

Resposta: The regression tables have been reformatted according to the recommendation. The reference category rows ("Ref.") were removed as separate lines, and only the Odds Ratios (OR) and their standard errors are now presented. Confidence intervals and p-values have been omitted from the table body, and p-values are indicated using the conventional asterisk notation (*p < 0.05; **p < 0.01; ***p < 0.001). Additionally, the crude (unadjusted) models have been moved to the statistical appendix, and only the adjusted models are presented in the main text to improve clarity and readability.

MINNOR COMMENTS

a) Page 2, line 62:
From: "Subsequently, the Odds/Odds Ratio and their respective 95% confidence intervals"
To: "Subsequently, the Odds Ratios and their respective 95% confidence intervals"

Resposta: The text has been corrected as suggested.

b) Page 3, line 81:
Please clarify what is meant by "distinct patterns," as the results across the waves do not appear markedly discrepant. Consider rephrasing to reflect better the observed differences, which seem subtle rather than pronounced.

Resposta: The sentence has been revised for clarity and accuracy. It now reads: "In conclusion, we observed variations in hospitalization patterns across pandemic waves, although the differences between them were subtle. These variations reflect gradual changes in risk factors associated with prolonged hospital stays."

c) Page 5, line 138:
From: The adopted inclusion criterion was confirmation of the diagnosis for COVID-19 19 with laboratory confirmation by RT-PCR ...
To: The adopted inclusion criterion was confirmation of the diagnosis for

COVID-19 with laboratory confirmation by RT-PCR ...

Resposta: The text has been corrected as suggested.

d) Page 14, line 242
From: the likelihood of prolonged hospitalization in elderly patients
To: the chance of prolonged hospitalization in elderly patients

Resposta: The text has been corrected as suggested.

d)Page 20, line 292-300:
Consider expanding the discussion regarding the vulnerabilities of older adults. While the current text highlights their susceptibility to psychological effects, it is important to emphasize that older adults are also at greater risk for a wide range of physical health complications. Beyond mental health issues, elderly individuals often experience other deteriorating body functions that may exacerbate COVID-19 outcomes. Deepening this analysis would provide a more comprehensive understanding of why age is consistently associated with prolonged hospitalizations and worse clinical outcomes.

Resposta: We have addressed this comment by adding two new paragraphs at the end of the Discussion section, elaborating on the broader vulnerabilities of older adults beyond psychological effects. The revised text emphasizes their increased risk for multiple physical health complications, the impact of comorbidities, and age-related physiological decline that may exacerbate COVID-19 outcomes. Three additional references were included to support this expanded discussion.

Yours Sincerely,
The authors
